# Nitric Oxide Responsive Heavy Metal-Associated Gene *AtHMAD1* Contributes to Development and Disease Resistance in *Arabidopsis thaliana*

**DOI:** 10.3389/fpls.2016.01712

**Published:** 2016-11-21

**Authors:** Q. Muhammad Imran, Noreen Falak, Adil Hussain, Bong-Gyu Mun, Arti Sharma, Sang-Uk Lee, Kyung-Min Kim, Byung-Wook Yun

**Affiliations:** ^1^Laboratory of Plant Functional Genomics, School of Applied Biosciences, Kyungpook National UniversityDaegu, South Korea; ^2^Department of Agriculture, Abdul Wali Khan UniversityMardan, Pakistan; ^3^Laboratory of Plant Molecular Breeding, School of Applied Biosciences, Kyungpook National UniversityDaegu, South Korea

**Keywords:** *R-*gene mediated resistance, basal defense, HR response, nitrosative stress, RNA-seq profile

## Abstract

Exposure of plants to different biotic and abiotic stress condition instigates significant change in the cellular redox status; resulting in the elevation of reactive nitrogen species that play signaling role in mediating defense responses. Heavy metal associated (HMA) domain containing genes are required for spatio-temporal transportation of metal ions that bind with various enzymes and co-factors within the cell. To uncover the underlying mechanisms mediated by *AtHMA* genes, we identified 14 *Arabidopsis HMA* genes that were differentially expressed in response to nitrosative stress through RNA-seq analysis. Of those 14 genes, the expression of eight HMA genes was significantly increased, whereas that of six genes was significantly reduced. We further validated the RNA-seq results through quantitative real-time PCR analysis. Gene ontology analysis revealed the involvement of these genes in biological processes such as hemostasis and transport. The majority of these nitric oxide (NO)-responsive *AtHMA* gene products are carrier/transport proteins. *AtHMAD1* (At1g51090) showed the highest fold change to *S*-nitrosocystein. We therefore, further investigated its role in oxidative and nitrosative mediated stress conditions and found that AtHMAD1 has antagonistic role in shoot and root growth. Characterization of *AtHMAD1* through functional genomics showed that the knock out mutant *athmad1* plants were resistant to virulent *Pseudomonas syringae* (DC3000) and showed early induction and high transcript accumulation of pathogenesis related gene. Furthermore, inoculation of *athamd1* with *avirulent* strain of the same bacteria showed negative regulation of *R*-gene mediated resistance. These results were supported by hypersensitive cell death response and cell death induced electrolyte leakage. AtHMAD1 was also observed to negatively regulate systemic acquired resistance SAR as the KO mutant showed induction of SAR marker genes. Overall, these results imply that NO-responsive *AtHMA* domain containing genes may play an important role in plant development and immunity.

## Introduction

Nitric oxide (NO), one of the smallest diatomic molecules is a highly reactive gaseous free radical and is the center of attention for most of the plant scientists due to its marvelous regulatory role in both plants and animals (Tuteja et al., 2004). Its high diffusivity (4.8 × 10^-5^ cm^2^ S^-1^ in H_2_O) allow it to move easily in various parts of the cell like cytoplasm and lipid layer of membranes (Arasimowicz and Floryszak-Wieczorek, 2007). A number of functions for NO in plants have been reported so far. This includes its regulatory role during salinity (Valderrama et al., 2007), drought (Zhao et al., 2004) heat (Rizhsky et al., 2004), cold (Beligni and Lamattina, 1999), and UV-B radiation (Shi et al., 2005). Due to its contribution NO was named as “Molecule of the Year” by the journal *Science* (Culotta and Koshland, 1992). Plants continuously interact with the external environment (biotic and/or abiotic) for multiple purposes. This interaction may range from positive such as with pollinators to negative such as with pathogens and herbivores (van Dam, 2009). Two major players of the negative interaction, biotic and abiotic stresses are the prime challenges for plants. These stresses lead to the production of increased reactive oxygen species (ROS) such as hydrogen peroxide, superoxide and hydroxyl radicals in the cell (Sudhakar et al., 2001; Xiong et al., 2002). Another type of important molecules produced during stress responses are reactive nitrogen species (RNS) (Delledonne et al., 1998) that has been shown to regulate a plethora of physiological activities and plant functions including plant defense and development (Delledonne et al., 1998; Durner et al., 1998). Most of these are formed as chemical derivatives of NO, primarily because NO itself is histologically toxic, short lived, and almost instantly reacts with other molecules. *S*-nitrosocysteine (CysNO) and *S*-nitrosoglutathione (GSNO) are considered important NO donors as they spontaneously decompose in aqueous solutions to release NO (Uehara et al., 2006; Cho et al., 2009). CysNO infiltrates into the plant cells via L-type amino acid transporters (Latson, 2011) resulting in *S*-nitrosylation of cellular proteins (Satoh et al., 1997; Zhu et al., 2008). The cell surface associated protein disulfide isomerase has been reported to aid in the incorporation of NO or nitroso moieties into the cell (Zai et al., 1999; Shah et al., 2007). Reduced Glutathione (GSH) is one of the most abundant low molecular weight thiols in the cell that contains glutamine, glycine, and cysteine residues. Compelling evidence about the role of GSH in gene expression, signal transduction, apoptosis, and NO metabolism has surfaced during the last decade (Pastore et al., 2001; Pompella et al., 2003). Furthermore, GSH is naturally used in combination with available NO to from GSNO, in a reversible reaction. Thus, it may play roles of both NO reservoir and NO donor (Stamler et al., 1992; Lindermayr et al., 2005). The enzyme GSNO reductase (GSNOR) is the primary enzyme that controls intracellular levels of GSNO and *S*-nitrosylated proteins in eukaryotes through de-nitrosylation (Feechan et al., 2005; Espunya et al., 2012).

The role of NO in biotic stress has also been studied. Sensing *avirulent* microbial pathogen activates the production reactive oxygen intermediates (ROIs) and reactive nitrogen intermediates (RNIs) (Burniston and Wilson, 2008) leading to the activation of multiple regulatory pathways. Similar to abiotic stress, plants have a specialized defense system to respond rapidly to pathogen ingress. In most cases virulent strains of bacterial pathogens like *Pst* can be recognized by *trans*-membrane pattern recognition receptors via pathogen-associated molecular patterns (PAMPs) such as flagellin (Zipfel and Felix, 2005). This activates PAMPs triggered immunity (Jones and Dangl, 2006). On the other hand, *avirulent* pathogens have effector proteins coded by *avr* genes that are recognized by the *R*-Proteins of the host plant activating *R*-gene mediated resistance (Jones and Dangl, 2006). Once the pathogen is successfully recognized, the production of reactive species, superoxides and hydrogen peroxide (H_2_O_2_) starts at the site of infection (Apostol et al., 1989; Grant and Loake, 2000). Such an interaction between *avr* and *R* gene products results in the effector-triggered immunity (ETI) (Jones and Dangl, 2006) is often associated with localized plant cell death, also known as the hypersensitive response (HR) and generation of systemic signals to activate systemic acquired resistance (SAR) (Ryals et al., 1996). Though the mechanistic control of *R-avr* interactions is still being investigated, the requirement of salicylic acid (SA) has been shown in many plant-pathogen interactions (MauchMani and Slusarenko, 1996). The phyto-hormones SA, jasmonic acid (JA), and ethylene play crucial roles in plant immunity by transmitting signals and inducing expression of specific genes (Feechan et al., 2005; López et al., 2008). An important feature of the plant defense response following pathogenic attack is the hypersensitive cell death response, an intentional suicide of cells at the site of infection to prevent the spread of disease (Jabs et al., 1996; Greenberg and Yao, 2004). Yun et al. (2011) reported that *AtGSNOR1* controls the level of nitrosothiol (SNO) in plants during the HR. They further suggested that in *atgsnor1-3*, SNO accumulation and SA production are inversely proportional; thus, the higher the level of SNO accumulation, the lower the production of SA and its β-glucoside derivative (SAG).

Metals are imperative in cellular and subcellular functions such as hydrolysis, oxidation of various molecules, and electron transfer. According to one estimate, one-third to half of all proteins require metallic ions, either as co-factors or as important structural components (Huffman and O’Halloran, 2001; Dupont et al., 2010).

Metallochaperones are specialized proteins responsible for the safe, site-specific transportation of metallic ions inside the cell (Huffman and O’Halloran, 2001; Haas et al., 2009; Robinson and Winge, 2010). For instance, heavy metal-associated isoprenylated plant proteins (HIPPs) that are metallochaperones contain a metal binding domain and play an important role in the transport of metal ions (Freund et al., 2008). The concept of metallochaperones was initially derived from the study of the CopZ protein and antioxidant protein 1 (ATX1) in bacteria and yeast, respectively. These small proteins (69 and 73 residues, respectively) have the Cys-XX-Cys metal binding motif within the first loop of a ferredoxin-like structural fold (βαββαβ) that represents the canonical heavy metal-associated domain (Freund et al., 2008; Robinson and Winge, 2010). These Cu metallochaperones have the ability to reduce the energy limit for the transfer of Cu to target proteins (Wernimont et al., 2000; Rubino and Franz, 2012).

Copper also plays a vital role in plant defense to pathogens. A good example is the interaction between rice and *Xanthomonas oryzae* pv. *oryzae* (*Xoo*), which causes bacterial blight disease. *Xoo* is sensitive to Cu; however, it has the ability to secrete an effector molecule that in turn activates transcription of the rice susceptibility gene *XA13*. This further induces Cu removal from xylem vessels of the infected plant, by promoting its binding with copper transporters COPT1 and COPT5. This, in turn, facilitates the spread of *X*. *oryzae* pv *oryzae* through plant vessels. This has been proven through the reverse genetics approach and a recessive allele of *XA13* has been found to confer increased resistance to *X*. *oryzae* pv *oryzae* infection (Freund et al., 2008). HMA domain containing genes are also reportedly involved in the interaction between plants and fungi. An example is *OsHIPP05* (*Os04g0401000*) that shows susceptibility to virulent *Magnaporthe oryzae*, while showing enhanced resistance to the avirulent race of the same pathogen (Fukuoka et al., 2009; Nakao et al., 2011). Another study showed that overexpression of rice HIPP05 makes the non-host plant *Arabidopsis* susceptible to *M. oryzae* (Nakao et al., 2011).

Metallic ions containing proteins can also play a role in transferring NO bioactivity. The nitrosylation of metallo-co-factors is considered mostly NO dependent rather than GSNO dependent (Foster et al., 2009; Yun et al., 2016) indicating the importance of NO in post-translational modifications (Foster et al., 2009; Yun et al., 2016). Furthermore, the key player of the SA signaling pathway in plants, the transcriptional co-activator NPR1, has been reported to bind with transition metals via Cys residues at its C-terminus (Wu et al., 2012). Therefore, the interaction of NO with bound transition metals might regulate *NPR1* during redox changes (Tada, 2009).

Although various roles of heavy metal associated (HMA) domain containing proteins have been described, however, little information is available about their roles in the protection of plants against pathogens and environmental stresses. In the present study, we adopted a combined *in silico* and biological approach to examine structure and putative function of the *Arabidopsis thaliana* HMA domain containing gene (*AT1G51090-AtHMAD1*) that showed differential expression in response to CysNO in a separate study involving transcriptomic analysis (Hussain et al., 2016). We also sought to determine its possible role in the regulation of oxidative and nitrosative stress and plant defense against virulent and avirulent pathogens. Collectively, these findings will lay a foundation for future studies on HMA domain containing proteins and will uncover their role in plant growth and defense.

## Materials and Methods

### *In silico* Data Analysis of HMA Domain Containing Genes

All the *A. thaliana* HMA (*AtHMA*) domain-containing genes that showed differential expression toward CysNO-mediated transcriptome analysis (Hussain et al., 2016) were identified and heat map showing the signal intensities of differentially expressed genes (up and down-regulated) was generated using R (version 3.3.1). Sequences for all *AtHMA* genes were retrieved from the *Arabidopsis* Information Resource (TAIR) database^1^. All sequences were aligned by the neighbor-joining method using ClustalW in BioEdit v7.2.5 (Hall, 1999). Promoter analysis of 1.5-kb region upstream of the transcription initiation site was also conducted using PlantCare (Lescot et al., 2002) to identify *cis*-regulatory elements. *Cis*-elements were then mapped using the Regulatory Sequence Analysis Tool (Medina-Rivera et al., 2015). Gene interaction analysis was performed using the protein–protein interaction network STRING (version 10.0^2^). We also performed Gene Ontology (GO) analysis to determine putative involvement of NO-responsive AtHMA proteins in biological processes, molecular function and their protein class through the GO consortium database^3^ using *A. thaliana* as reference genome (Mi et al., 2013). The results so obtained at *P*-value < 0.05 were selected and compiled into pie charts.

### Plant Material and Growth Conditions

Seeds of the *Arabidopsis* (Col-0) *athmad1* (*AT1G51090*) loss of function mutant were obtained from Nottingham Arabidopsis Stock Centre (NASC)^4^ and grown either on 1/2 Murashige and Skoog (MS) medium or soil at 23 ± 2°C, under long day conditions (16 h light and 8 h dark). All plants were genotyped at the rosette leaf stage (4 weeks old plants unless stated otherwise) in order to identify homozygous lines. *Arabidopsis* wild type (WT) and all other lines used in the study were of the Col-0 genetic background. Perturbations in the cellular levels of ROS and RNS levels affect metal-containing enzymes such as peroxidases and catalases (Clarke et al., 2000; Fourcroy et al., 2004). Since our study included experiments to determine the role of HMA containing genes in oxidative and nitrosative stress in plants, we used the *Arabidopsis gsnor1-3* mutant as a comparative control.

### Redox Stress Assay

*Arabidopsis athmad1* plants were tested for various growth parameters under oxidative and nitrosative stress conditions, as described by Sharma et al. (2016). *Arabidopsis* WT and *athmad1* seeds were surface sterilized and germinated on 1/2 strength MS medium in at least three replicates each, containing 2 mM H_2_O_2_, 1 μM methyl viologen (Medina-Rivera et al., 2015), 0.75 mM *S*-nitrosoglutathione (GSNO), and 0.75 mM *S*-nitrosocysteine (CysNO). Data on hypocotyl emergence, cotyledon development frequency (CDF), and root and shoot length were recorded 1 week after treatment.

### Pathogen Inoculation

Virulent *Pseudomonas syringae* pv. *tomato* (Jaffrey et al., 2001) strain DC3000 and *avirulent Pst* DC3000 expressing the *avrB* effector were grown, maintained, and inoculated, as described previously (Yun et al., 2011). Briefly, both the bacterial strains were cultured on LB (Luria-Bertani)-agar media with appropriate antibiotics for selection (100 μg/mL rifampicin for virulent *Pst* DC3000 and kanamycin [50 μg/mL] and rifampicin for *Pst* DC3000 [*avr*B]), following which a single colony was transferred to LB broth and incubated at 28°C overnight. Both bacterial strains were then harvested by centrifugation at 13,000 rpm for 1 min in 10 mM MgCl_2_, and syringe-infiltrated into the abaxial side of leaves at a concentration of 5 × 10^5^ CFU (unless stated otherwise). Control plants were only infiltrated with 10 mM MgCl_2_. Plants were observed regularly for disease symptoms and leaf samples were collected for further analysis.

### Pathogenicity Assessment and Electrolyte Leakage Assay

Leaf samples (1 cm each) from plants inoculated with *Pst* DC3000 were collected, crushed in 1 mL of sterile 10 mM MgCl_2_ 2 and 4 days post inoculation (DPI). The homogenate was diluted 10 times and spread on LB-agar plates containing appropriate antibiotics. Plates were incubated at 28°C for 48 h and colonies were counted. Ion or electrolyte leakage was measured as described by Dellagi et al. (1998) with some modifications. Conductivity was measured 0, 1, 2, 4, 6, 8, 10, 12, and 24 h post infiltration (hpi), using a portable conductivity meter (HURIBA Twin Cond B- 173, Japan).

### Quantitative Real-Time PCR (qRT-PCR) Analysis

RNA was extracted from the inoculated plants using Trizol^®^ (Invitrogen, USA) as described by Imran et al. (2016). For gene expression analysis, cDNA was synthesized using the DiaStar^TM^ RT kit (SolGent, Korea) according to the manufacturer’s instructions. PCR was performed in the EcoTM real-time PCR machine (Illumina, USA) using 2X Quantispeed SYBR Kit (PhileKorea) along with 100 ng of template DNA and 10 nM of each primer in a final volume of 20 μL. A “no template control” was used as a negative control that contained distilled water instead of template DNA. Two-step PCR reactions were performed for 40 cycles using primers given in Supplementary Table S1.

### Histological Staining

*Pseudomonas syringae* pv. tomato expressing the *avr*B effector proteins activate *R* gene-mediated (RPM1) defense in *A. thaliana*, thereby triggering hypersensitive resistance (Leister et al., 1996). The HR of *athmad1* plants to *Pst* DC3000 (*avr*B) infiltration was quantified via trypan blue staining as described by Koch and Slusarenko (1990). Cell death was quantified (arbitrary units) in terms of the intensity of trypan blue staining using Adobe Photoshop CS6^5^, as described earlier (Yun et al., 2011).

### SNO Measurement

As mentioned previously, published evidence suggests an inter-relationship between NO and metal biology in plants under basal, as well as induced conditions. *Arabidopsis atgsnor1-3* accumulates comparatively higher levels of SNO (Feechan et al., 2005). This prompted us to investigate the *S*-nitrosothiol levels in *athmad1* before and after inoculation with *Pst* DC3000 (*avrB*). For this purpose, *Arabidopsis* WT, *atgsnor1-3*, and *athmad1* plants were syringe- infiltrated with 5 × 10^5^ CFU mL^-1^ *Pst* DC3000 (*avrB*). Leaf samples were collected and ground in KPI buffer (pH 5.5) after 0, 6, 12, and 24 h post infiltration. SNO measurements were performed in an NO analyzer (NOA-280i, Sievers, USA).

### Foliar SA Application

For foliar application of SA, we used the method described by Gao et al. (2011) with minor modifications. Four-week-old soil grown *WT, atgsnor1-3*, and *athmad1* plants were uniformly sprayed with 1 mM SA. Control plants were sprayed with distilled water. Leaf samples were collected at 0, 12, and 24 h after spraying for further analysis.

### Statistical Analysis

All the data was statistically analyzed using analysis of variance followed by standard error through Student Statistics 9 software. Student *t*-test was performed using Microsoft Excel program.

## Results

### Validation of RNA-Seq Data through qRT PCR and GO-Enrichment Analysis

**FIGURE 1:**
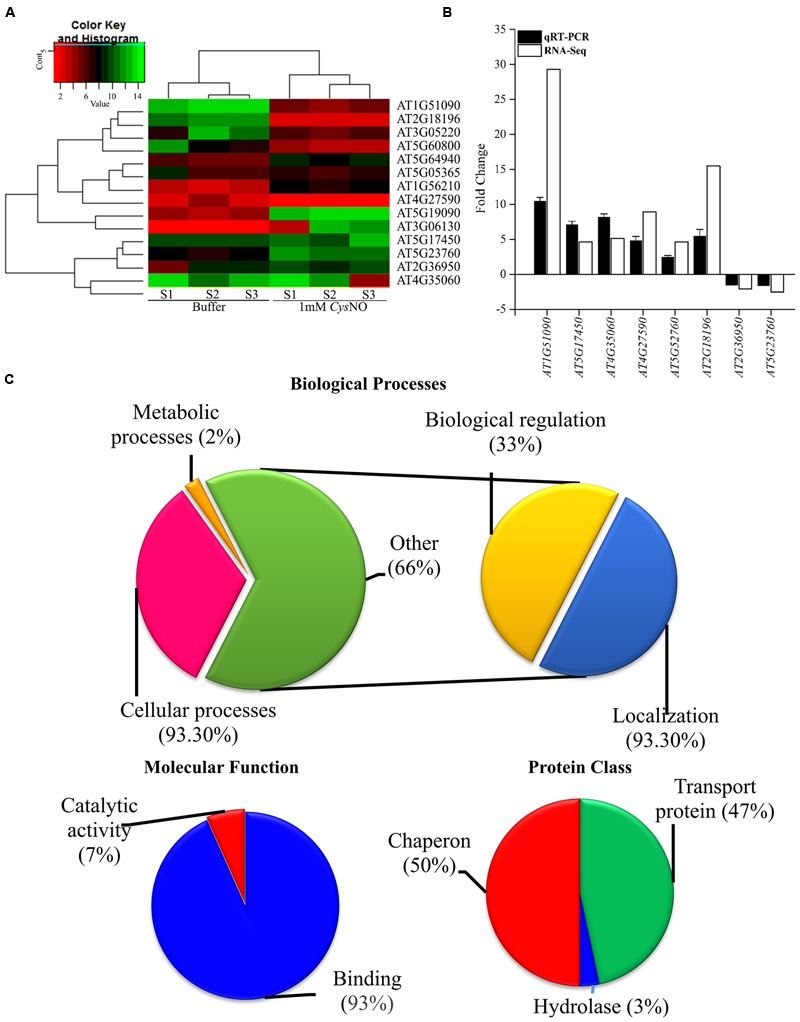
**NO-responsive heavy metal associated domain genes. (A)** Heat map of the *AtHMA* genes showing the signal intensities of differentially expressed genes in response to 1 mM CysNO through RNA-seq analysis. **(B)** Validation of RNA-seq data through qRT PCR analysis of randomly selected DEGs. **(C)** GO-enrichment analysis showing role of *AtHMA* genes in different biological processes and their classification into different protein classes.

The overall gene expression profile of *AtHMA* genes that showed differential response to CysNO through RNA-seq analysis (**Figure 1A**, Suplementary Table 2) were further validated through qRT PCR analysis (**Figure 1B**). All of the six randomly selected up-regulated HMA genes showed significantly high transcript accumulation, whereas two down-regulated genes showed significantly reduced transcript accumulation compared to buffer (**Figure 1B**). A correlation coefficient of 0.67 shows high congruence of RNA-Seq and qRT-PCR experiments. All these genes were then analyzed for GO terms of Biolgoical processes, Protein class and Molecular function usigng GO Consortium^6^. The results indicated that 93.30% of genes were involved in cellular processes, 2% in metabolic processes, 33% in biological regulation (Hemostasis), and 93.30 % are involved in localizaition [transport (**Figure 1C**)].

### Conserved Domain and Promoter Analysis for *Cis*-Regulatory Elements

Analysis for the presence of conserved motifs showed the presence of a highly conserved Cys-XX-Cys motif in 11 of the 14 gene products (Supplementary Figure S1E; blue box). Furthermore, the genes having Cys-XX-Cys also contained three highly conserved lysine (K) residues, and other important conserved residues (Supplementary Figure S1E).

**FIGURE 2:**
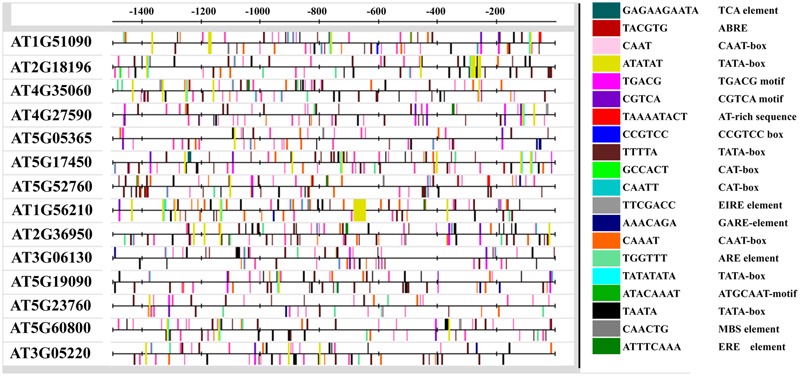
**Promoter analysis for the presence of *cis-*regulatory elements.** A total of 14 HMA domain containing genes were analyzed for the presence of *cis*-regulatory elements, 1.5 kb upstream of the transcription initiation site. The resultant *cis-*regulatory elements were mapped using Regulatory Sequence Analysis Tool (Shirsat et al., 1989) (http://floresta.eead.csic.es/rsat/) for better presentation.

*Cis*-regulatory elements are non-coding DNA sequences present in the promoters of various genes to which various regulatory proteins bind in order to control gene expression. These elements reportedly play a major role in transcriptional control of genes (Wittkopp and Kalay, 2012) involved in defense related pathways (Caarls et al., 2015) and plant responses toward pathogens (Rushton and Somssich, 1998). Therefore, we analyzed promoter sequences of all the *AtHMA* genes for the presence of *cis*-regulatory elements. We found 19 *cis*-regulatory elements in the promoters at variable distances form the transcriptional initiation site. These elements included ABRE (TACGTG) involved in regulation of osmotic and drought stress responsiveness genes (Kim et al., 2011); the CAAT-box (CAAT/CAAAT), responsible for tissue specific activity (Shirsat et al., 1989); AT-rich sequences (TAAAATACT) and EIRE (TTCGACC) involved in maximal elicitor-mediated activation (Fukuda, 1997); the TATA-box, a core promoter element for enhanced transcription (Mukumoto et al., 1993); ERE (ATTTCAAA) that are ethylene-responsive elements (Ohme-Takagi and Shinshi, 1995); and the MYB binding site (MBS; CAACTG) involved in drought-stress regulation (Singh and Laxmi, 2015), (**Figure 2**). This suggests the importance of AtHMA domain containing proteins in biotic and abiotic stress tolerance.

### Metallo-Protein Interaction

As AtHMA proteins are mostly involved in heavy metal transport (de Abreu-Neto et al., 2013); therefore, to determine the possible ligand-binding interactions of AtHMA proteins, we performed an *in silico* experiment to analyze the protein sequence of AtHMAD1 for its ability to bind various metal ligands using RaptorX (Kallberg et al., 2012) which is a web portal for protein structure and function prediction using protein sequences. Our results showed that the first domain (1–80 amino acids) of AtHMAD1 can bind to different metal ligands at different rates (Supplementary Figure S1D), as represented by pocket multiplicity (PM; the frequency at which a particular pocket was found in a set of ligand- protein structures). The highest PM was observed for Cu at sequence residues N15, C16, S17, and C19 with a PM rate above 40 (Kallberg et al., 2012). This suggests that AtHMAD1 might be involved in Cu (or other metal ligands) binding, which is a characteristic feature of metallochaperones. The strong *in silico* binding interaction suggests the possible role of AtHMAD1 in metal transportation in and out of the cells.

### AtHMAD1 Positively Regulates Shoot Growth and Negatively Regulates Root Growth under Oxidative and Nitrosative Stress Conditions

**FIGURE 3:**
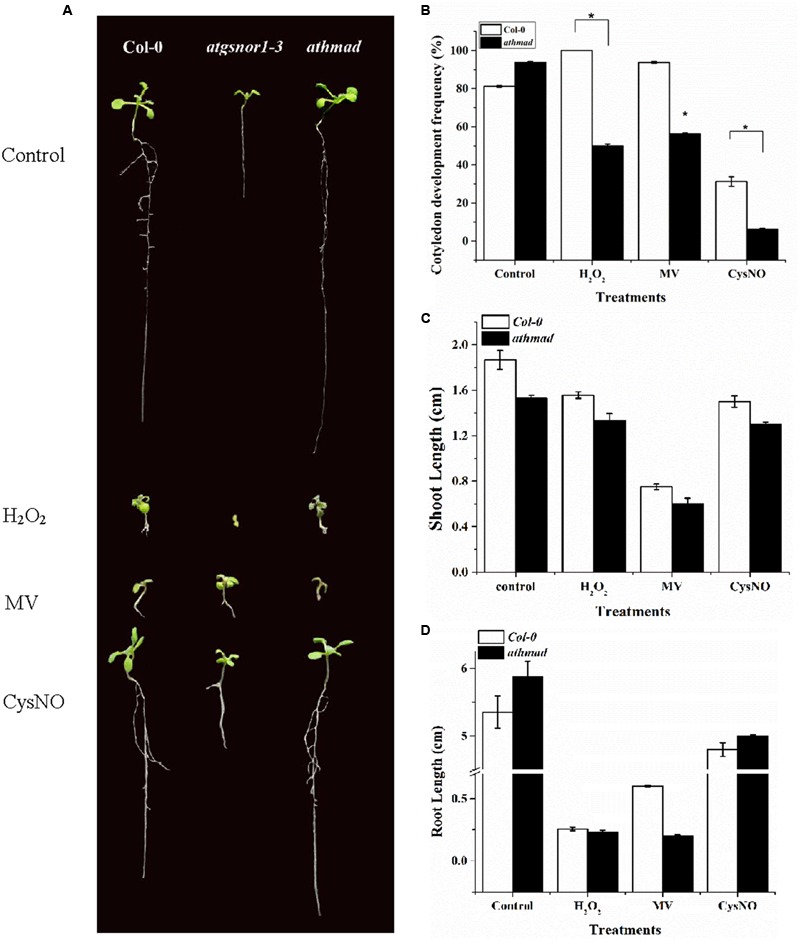
**Role of *AtHMAD1* in plant development. (A)** Phenotypes of the indicated genotypes under control and redox stress conditions. **(B)** In response to oxidative and nitrosative stress *athmad1* plants showed reduced CDF **(C)**, shoot length, and **(D)** root length compared to untreated control plants. All data points represent means of three replicates. The experiment was repeated three times with similar results. Error bars represent the standard error. Significant interactions are indicated by an asterisk (Student’s *t*-test with a 95% confidence level).

A number of growth parameters like hypocotyl emergence, CDF, shoot and root length in WT and loss-of-function mutant *athmad1* plants were studied to determine the possible role of AtHMAD1 in plant growth and development. CDF represents the number of developed, green seedlings with the first cotyledonary leaves (under nitrosative stress, seeds of several *Arabidopsis* genotypes may germinate but ultimately die, e.g., *atgsnor1*-3 and other NO-related genotypes; Yun et al., 2011). Under oxidative (2 mM H_2_O_2_, 1 μM methyl viologen,) and nitrosative stress (0.75 mM GSNO, and 0.75 mM CysNO), *athmad1* plants showed a significant reduction in hypocotyl emergence (Supplementary Figure S3), CDF (**Figure 3B**), and shoot length (**Figures 3A,C**) compared to WT. In contrast, *athmad1* plants showed an increase in root length under nitrosative stress; however, compared to WT, a significant reduction in root length was observed in *athmad1* plants under conditions of oxidative stress induced by H_2_O_2_ and MV (**Figures 3A,D**).

### AtHMAD1 Negatively Regulates Plant Basal Defense

**FIGURE 4:**
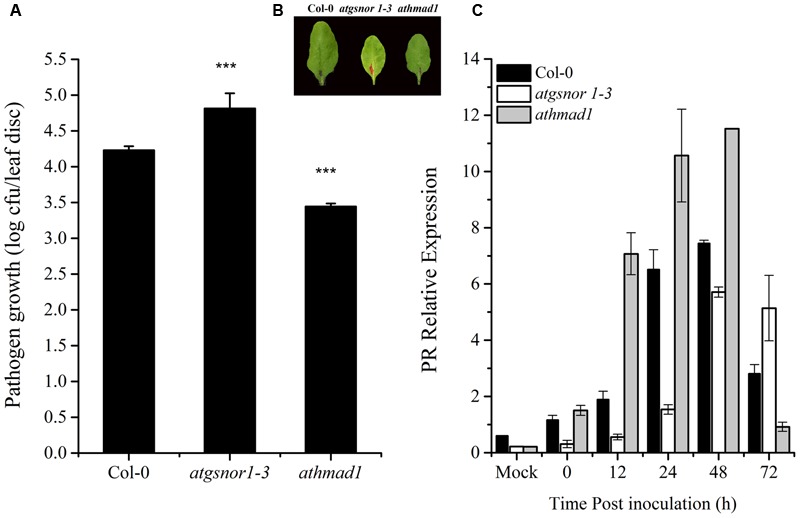
***AtHMAD1* negatively regulates the basal defense system.** Pathogen growth from the inoculated leaves with virulent *Pst* DC3000 at 5 × 10^5^ colony forming units (CFU) mL**^-^**^1^after 2 days post inoculation (DPI; **A)**. Development of symptoms in wild type (WT) and transgenic lines following infiltration with virulent *Pst* DC3000 **(B)**. Transcript accumulation of the *PR* gene (SA-dependent pathway) in the indicated *Arabidopsis* genotypes in response to pressure infiltration of *Pst* DC3000 using qPCR **(C)**. The expression values were normalized using the constitutively expressed actin. All data points represent the mean of four replicates. Error bars represent ± SE (*n* = 4) with asterisks indicate highly significant differences (99% confidence level) from WT plants. The experiment was repeated three times with similar results (Student’s *t*-test, *P* < 0.0000265).

To investigate whether AtHMAD1 is required for basal defense, we inoculated *athmad1* plants, as well as the *atgsnor1-3* and WT control plants with virulent *Pst* DC3000. The *atgsnor1-3* line that is reportedly susceptible to *Pst* DC3000 (Feechan et al., 2005) exhibited significantly higher levels of bacterial growth compared to WT plants. The *athmad1* plants showed significant resistance toward this pathogen compared to the WT plants (**Figures 4A,B**) suggesting that AtHMAD1 negatively regulates plant defense. This was further confirmed by rapid and high levels of transcript accumulation of the plant defense marker gene *PR1* following pathogen inoculation, as evidenced by qRT-PCR analysis (**Figure 4C**).

We also determined whether induction of *PR* genes would have an effect on the expression of *PDF1.2* in *athmad1* plants following inoculation with virulent *Pst* DC3000. *PDF1*.2 is a marker gene for JA pathway. Literature reports suggested a negative interaction between SA and JA pathway (Penninckx et al., 1996; Niki et al., 1998). Therefore, we sought to determine interaction between SA and JA pathway in *athmad1* mutants. Expression of *PDF1.2* in *athmad1* plants was observed to be significantly lower than that of WT plants (Supplementary Figure S4).

### AtHMAD1 also Negatively Regulates *R*-Gene Mediated Resistance

**FIGURE 5:**
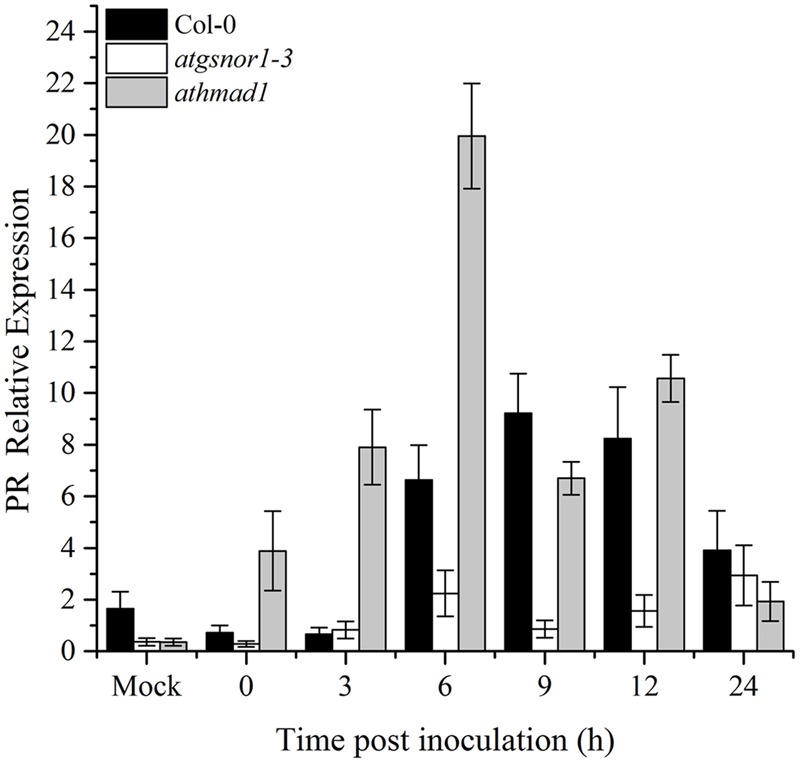
**Role of *AtHMAD1* in *R*-gene mediated resistance.** Early induction and high transcript accumulation of the *PR* gene in *athmad1* plants in response to *Pst* DC3000 (*avrB*) inoculation at 5 × 10^5^ colony forming units (CFU) mL**^-^**^1^ in the indicated *Arabidopsis* genotypes. All data points represent the mean of three replicates. All the data was analyzed statistically using analysis of variance (*P* < 0.05) followed by Standard Error. Error bars represent ± SE (*n* = 3). The experiment was repeated three times with similar results.

To determine if AtHMAD1 function is required for *R*-gene mediated resistance, we infiltrated plants with *Pst* DC3000 (*avrB)*. Our results indicated early induction and high transcript accumulation of the *PR* gene over time compared to WT (**Figure 5**). We also examined whether AtHMAD1 is involved in the JA defense pathway, by studying the expression level of the JA-dependent pathway gene *PDF1*.2. In contrast, *PDF1*.2 expression was significantly lower compared to WT (Supplementary Figure S5), indicating an antagonistic relationship between *PR* and *PDF1*.2 during plant defense responses.

### Pathogen-Triggered Hypersensitive Response (HR) Is Induced in *athmad1* Plants

**FIGURE 6:**
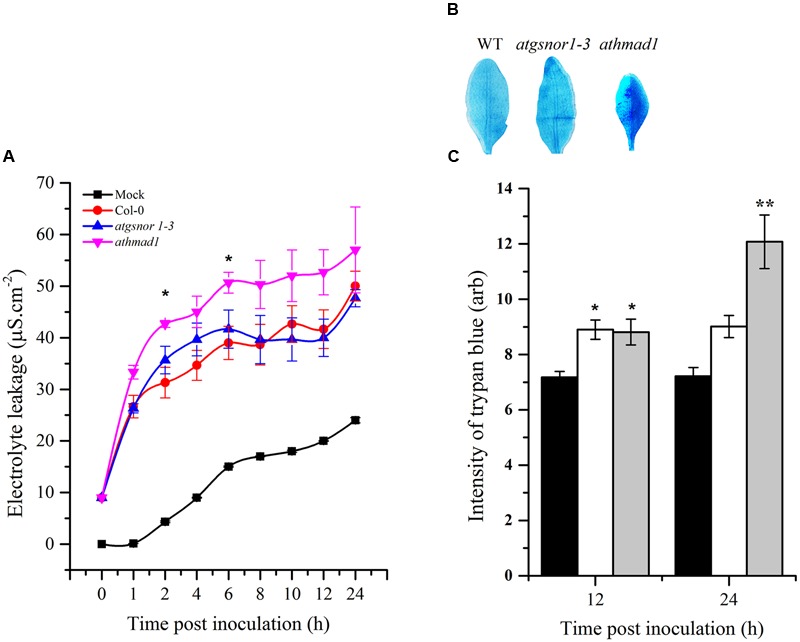
**Induced HR response of *athmad1* plants following attempted *Pst DC3000* (*avrB*) infection. (A)** Electrolyte leakage; **(B)** Visualization of cell death by trypan blue staining; and **(C)** Its quantification using the Luminosity tool of Photoshop by fixing the values for the rectangular marquee tool. Values represent the means of three random replicates. The error bars represent ± SE (*n* = 3), and asterisks represent significant differences compared to WT plants, (Student’s *t-*test with a 95% confidence level).

Effector-triggered immunity ultimately culminates in the hyper sensitive response (HR), that is, the intentional execution of plant cells at the site of infection. To identify the possible role of AtHMAD1 in HR induction, we challenged plants with avirulent *Pst* DC3000 (*avrB*). To visualize cell death *in vivo*, the inoculated leaves were stained with lactophenol trypan blue. An increase in trypan blue staining was observed which was used as a method to detect and quantify cell death. The *athmad1* plants showed an increased HR after 12 and 24 h of inoculation compared to WT plants (**Figure 6B**,), that was also confirmed by quantifying the cell death (**Figure 6C**) indicating that AtHMAD1 negatively regulates HR. For further confirmation, we quantified cell death induced electrolyte leakage. The *athmad1* plants showed higher levels of electrolyte leakage over time, followed by the *atgsnor1-3* plants with significant difference (*P* < 0.05) at 2 and 6 hpi compared to WT (**Figure 6A**).

### *athmad1* Plants Showed High Induction of SAR Associated Genes

**FIGURE 7:**
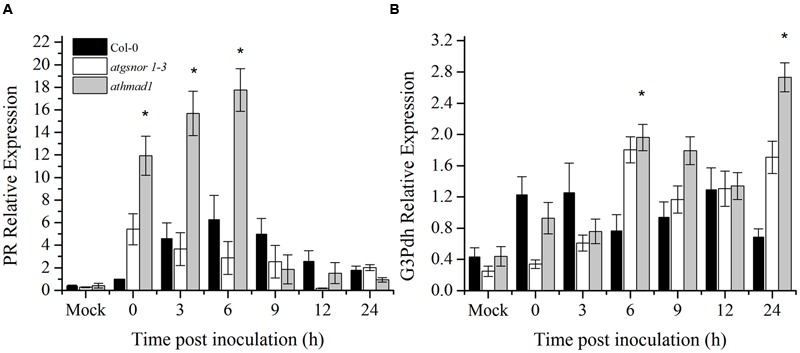
**Transcript accumulation of genes involved in SAR.** Expression level of **(A)** the *PR* gene and **(B)** *G3Pdh* (Glycerol-3-phosphate dehydrogenase) following attempted *Pst DC3000* (*avrB*) infiltration at 5 × 10^5^ CFU. Leaf samples were collected from systemic leaves at various intervals over 24 hpi. 0 hpi represents samples collected immediately after pathogen inoculation. The experiment was repeated three times with similar results. All the data was analyzed statistically using analysis of variance (*P* < 0.05) followed by Standard Error. Error bars represent ± SE (*n* = 3). Asterisks represent significant differences compared to WT plants, (Student’s *t-*test with a 95% confidence level).

A number of SAR associated signals have been identified so far. These include SA and its methylated derivative MeSA and azelaic acid (Park et al., 2007; Shah and Zeier, 2013; Wang et al., 2014; Wendehenne et al., 2014). To determine the role of AtHMAD1 in SAR activation, we studied the induction of *PR* and *G3Pdh* genes over time in systemic leaves following *Pst* DC3000 (*avrB)* inoculation. High induction of *PR* gene was observed in systemic leaves of *athmad1* plants at earlier time points (0, 3, and 6 hpi) compared to WT plants and *atgnsor 1-3* (**Figure 7A**). Furthermore, after 24 h of inoculation, high expression levels of *G3Pdh* (the detail description about this gene is given in Discussion section) was observed in systemic leaves of *athmad1* plants in comparison to those of WT plants (**Figure 7B**). Whereas transcripts of *PDF1.2* were significantly reduced in *athmad1* plants compared to WT plants (Supplementary Figure S6), indicating that AtHMAD1 negatively regulates SAR.

### Exogenous Application of SA Induces PR Gene, but Does Not Induce SAR-Associated Genes in *athmad1* Plants

**FIGURE 8:**
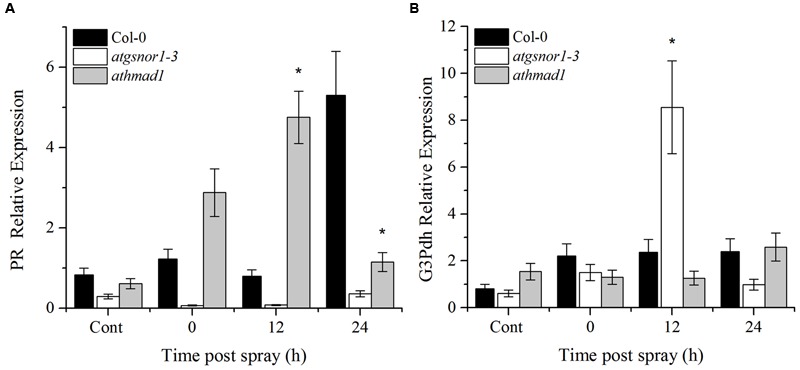
**Transcriptional analysis of exogenous SA application. (A)** *PR* gene expression; and **(B)** *G3Pdh* (Glycerol-3-phosphate dehydrogenase) expression in response to 1 mM SA using RT-qPCR. The corresponding values were calculated relative to those of the constitutively expressed Actin gene. All the data was analyzed statistically using analysis of variance (*P* < 0.05) followed by Standard Error Error bars represent ± SE (*n* = 3). Asterisks represent significant differences compared to WT plants, (Student’s *t-*test with a 95% confidence level).

An early induction and high expression of the *PR* gene was observed in *athmad1* plants compared to WT and *atgsnor1-3* plants, following the application of SA spray (**Figure 8A**). On the other hand, *G3Pdh* expression was not induced with exogenous application of SA in *athmad1* plants compared to WT (**Figure 8B**), indicating that AtHMAD1 may interact with SAR upstream of *G3Pdh*. Another reason for this might be that the NO and *G3Pdh* pathway is distinct from the SA pathway, as described by El-Shetehy et al. (2015), who reported that exogenous SA is unable to restore SAR in ROS- or NO-deficient mutants.

### Pathogen-Triggered SNO Accumulation Was Reduced in *athmad1* Plants Following the Application of SA Spray

**FIGURE 9:**
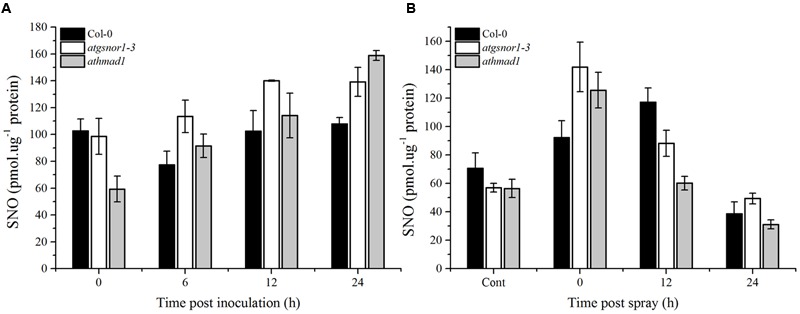
**Accumulation of *S*-nitrosothiol (SNO) in response to (A)** *Pst* DC3000 (*avrB*) inoculation and **(B)** exogenous SA application. The indicated genotypes were infiltrated with *Pst* DC3000 (*avrB*) or 1mM SA. control plants were sprayed with distilled water. The total level of cellular SNO was determined over time using NO analyzer (NOA-280i, Sievers, USA). Error bars represent ± SE (*n* = 3).

The major route of NO signaling in plants is considered a direct one, involving *S*-nitrosylation to form SNOs. We observed a gradual increase in SNO accumulation in *athmad1* leaves after 6, 12, and 24 h of inoculation with the pathogen, and this difference was most significant after 24 h (**Figure 9A**). We then sprayed plants with 1 mM SA to examine the interaction between AtHMAD1 and SA during SNO accumulation in plants. SNO content was significantly reduced at 12 and 24 h following the application of SA spray in *athmad1* plants, compared to WT control plants (**Figure 9B**). Published reports suggest that SA positively regulates S-nitrosoglutathione reductase (*GSNOR*) (Feechan et al., 2005). Thus, a reduction in SNO contents following exogenous application of SA might be due to SA-mediated *GSNOR* induction that reduced the elevated NO levels within the cell.

## Discussion

Heavy metal associated domain containing proteins have been shown to play a pivotal role in plant growth and stress perception (Williams et al., 2000; de Abreu-Neto et al., 2013). HMA domain containing genes not only serve as metal ion transporters for different essential functions and/or detoxification, but are also important reserviors of essential metal ions that are required for growth and development (Williams et al., 2000; Clemens, 2001; Grotz and Guerinot, 2006). By using a variety of metal-associated loss of function mutants and/or over-expressor transgenic genotypes, a number of published functional genomic studies have shown the importance of metal biology in plant growth and development in the model plant *Arabidopsis* (Dykema et al., 1999; Barth et al., 2009; Tehseen et al., 2010), as well as various cultivated crops such as soybean, tobacco (Dykema et al., 1999), and rice (de Abreu-Neto et al., 2013). Both ROIs and RNIs regulates metal ion homeostasis in plants (Burniston and Wilson, 2008). Heavy metal-associated domain containing genes are metallochaperones that have the HMA domain Cys-XX-Cys metal ion binding motif within the first loop of a ferredoxin like structural fold (βαββαβ) that is mainly responsible for the delivery of metal ions within the cell (Robinson and Winge, 2010).

Under normal conditions, plants produce basal levels of NO that usually increase in response to biotic and/or abiotic stresses. By utilizing high throughput transcriptomic analysis, we identified a list of 14 genes that showed differential expression in response to 1 mM CysNO (Supplementary Table S2). The use of spatiotemporally inducible promoters is considered important for increased resistance to plant pathogens. The expression patterns and regulation strategies of only a few plant promoters induced in response to phyto-pathogens have been characterized so far (Gurr and Rushton, 2005). Furthermore, besides the core promoter area, other auxiliary transcription regulatory elements like enhancers, silencers, and insulators can be found at 5′ upstream and 3′ downstream of the promoter region and introns (Cawley et al., 2004). The promoter analysis for *cis*-regulatory elements in *AtHMA* domain containing genes indicated the presence of various important motifs and elements that regulate gene expression under various environmental conditions and during pathogen ingress (**Figure 2**). For instance, the presence of ABRE that is involved in ABA responses; ERE, that are ethylene responsive elements; and EIRE, involved in maximal elicitor-mediated activation, suggests the possible role of *AtHMA* domain containing genes in drought tolerance, growth, and defense against various pathogens (**Figure 2**).

Structure prediction and domain binding analysis of AtHMAD1 suggest its possible role in metal binding and/or transport, thus regulating various physiological and molecular processes under biotic and abiotic stress. AtHMAD1 showed high PM for Cu, an indication of a true binding interaction (Supplementary Figures S1A-D). Interestingly, Cu is the co-factor for important enzyme nitrite reductase that catalyzes the formation of NO through a reversible reaction with ferrocytochrome-C in the presence of two hydrogen protons thus controlling the cellular RNS levels. In addition, it is also a co-factor for other important enzymes like galactose oxidase, 2-furoyl-CoA dehydrogenase, nitrous oxide reductase, NO reductase, cytochrome-c oxidase, tyrosinase, etc^7^. These results suggests the putative regulatory role of AtHMAD1 in the regulation of enzyme function during important cellular processes, particularly under stressful conditions.

Gene interaction studies of *AtHMAD1* (AT1G51090) yielded interesting information about its association with other genes (Supplementary Figure S2). Its strong association with Gibberellin 2-Oxidase 6 (*GA2ox6*:AT1G02400) predicts the importance of this gene in plant growth and development. We observed reduced hypocotyl emergence, CDF, and shoot growth (**Figures 3A–C**) under conditions of oxidative and nitrosative stress in *athmad1* plants compared to WT. This reduction could be attributed to excess accumulation of ROS and RNIs, in response to changes in the redox environment as described by García-Mata and Lamattina (2001), Uchida et al. (2002), and Kopyra and Gwóźdź (2003). Altogether, these results imply that the AtHMAD1 gene product negatively regulates CDF and shoot growth.

The *athmad1* loss of function mutant showed greater resistance to the virulent pathogen *Pst* DC3000 compared to WT plants (**Figure 4**). On the other hand, the susceptible mutant *atgsnor1-3* showed significantly higher bacterial growth. This is because of the already established fact that *S*-nitrosoglutathione reductase (GSNOR) is required for both basal and *R*-gene mediated resistance (Feechan et al., 2005). Studies reveal that cell death occurs mainly because of ROIs and RNIs that are produced by NADPH oxidase and nitrosative bursts, respectively (Delledonne et al., 1998; Yun et al., 2011). Early induction and high transcript accumulation of the PR gene in response to *Pst* DC3000 in the *athmad1* mutant compared to WT suggests that AtHMAD1 protein regulates plant defense through the SA-dependent pathway.

Plant defense starts with the recognition of PAMPs by plant pattern recognition receptors (PRRs), resulting in PAMP-triggered immunity (PTI). Some pathogens have evolved to evade PTI by deploying certain effector molecules into the host system, thereby leading to effector-triggered susceptibility (Jones and Dangl, 2006). However, different effector molecules are recognized by plant disease resistance (*R*) genes encoded by the nucleotide binding site leucine rich repeat (NBS-LRR) genes that recognize these effector molecules (Jones and Dangl, 2006). Published reports suggest that following pathogenic infection, SA accumulation is responsible for the induction of *PR* genes (Durrant and Dong, 2004). ETI culminates with HR. Therefore, to investigate the possible role of AtHMAD1 in ETI, we also examined the HR following inoculation with *Pst* DC3000 (*avrB*). The *athmad1* plants displayed enhanced pathogen-triggered cell death and early induction of *PR* gene expression compared to WT plants (**Figures 5** and **6B,C**). Earlier reports suggest that *R* gene-mediated resistance is conferred because of interaction between *avr* and *R*-gene products that serves to limit the growth of virulent pathogens inside the host plant (Glazebrook et al., 1996; Parker et al., 1996). A similar study has been conducted in rice showing that avirulent strain of *X. oryzae* pv. *oryzae* which show sensitivity to Cu, release effector molecules that induces rice susceptibility gene *XA13*. This further triggers Cu removal from xylem vessels by inducing its ability to bind with COPT1 and COPT5 which are copper transporter proteins thus facilitating the spread of *X. oryzae*. The study has been validated through reverse genetics studies and it was suggested that recessive allele of XA13 confers enhanced resistance to *X. oryzae* pv *oryzae* infection (Freund et al., 2008). Our results suggested reduced transcript accumulation of *PDF1*.*2* in *athmad1* plants compared to WT plants after attempted inoculation of both *Pst* DC3000 and *Pst* DC3000 (*avrB*). This is consistent with the findings of Niki et al. (1998) who described the antagonistic effects of SA and JA during regulation of defense pathways. It was also reported that *PDF1*.2 showed high levels of expression in the SA-deficient mutant NahG (Penninckx et al., 1996). Therefore, low levels of transcript accumulation of *PDF1*.2 in samples showing increased *PR* gene expression indicates negative interaction between the two immune activators. The notion that AtHMAD1 modulates the SA-dependent pathway can also be supported by the levels of SNO observed in *athamd1*. Our results suggests that after *Pst* DC300 (*avrB*) inoculation, a gradual increase in SNO levels was observed in *athamd1* plants compared to WT (**Figure 9A**), however, after SA spray the SNO levels decreased over time in *athmad1* compared to WT (**Figure 9B**). Earlier reports suggested that high levels of SNO were observed in *nox*-*1* and *atgsnor1*-3 plants that blunted the SA-dependent pathway (Feechan et al., 2005; Yun et al., 2016). In contrast, *atgsnor1*-*1* and *atgsnor1*-2 plants showed increased SA levels and reduced basal SNO levels (Feechan et al., 2005). Cellular NO levels can be regulated via the NO donor, GSNO that can be reduced to GSSH (Glutathione disulfide) and NH_3_ in the presence of *GSNOR*. Thus, a reduction in SNO contents following exogenous application of SA might be due to SA-mediated *GSNOR* induction that reduced the elevated NO levels within the cell.

Systemic acquired resistance is another important plant defense system induced by primary infection. It provides broad spectrum disease resistance and protects other parts of the plant from secondary infections (El-Shetehy et al., 2015). Earlier reports have shown that NO and ROS are required for activation of SAR (Alvarez et al., 1998; Song and Goodman, 2001). The accumulated evidence suggests that a number of SAR signals act to confer resistance in distant tissues to subsequent infections (El-Shetehy et al., 2015). Azelaic acid (AzA) and Glycerol 3 phosphate (G3P) are important SAR signaling molecules (Yu et al., 1999, 2013). Exogenous application of AzA increases the expression of G3P biosynthesis genes, GLY1 and GLI1 that encodes G3P dehydrogenase and glycerol kinase, respectively. Reports suggested that AzA and G3P-induced signaling was connected somehow in the SAR pathway. Recently, a study by Yu et al. (2013) suggested that SAR inducer AzA conferred SAR in wild type but not in plants defective in the G3P biosynthesis genes GLI1 and G3Pdh (Chanda et al., 2011). Furthermore, G3P levels were also significantly increased after AzA application which was consistent with increased G3Pdh expression suggesting that AzA-mediated SAR require G3Pdh gene (Yu et al., 2013). Therefore, we also studied the AtHMAD1 role in SAR signaling by studying the expression of marker genes (PR and G3Pdh) (Wang et al., 2014) both in local and systemic leaves. High induction of PR and G3Pdh genes was observed in systemic leaves of *athmad1* plants overtime compared to WT suggesting negative role of ATHMAD1 in SAR.

Together all these results suggest that AtHMAD1 gene product negatively regulates multiple modes of disease resistance such as basal defense, *R*-gene mediated resistance, hypertensive cell death response, and systemic acquired disease resistance. Furthermore, it negatively regulates shoot growth while positively regulates root growth under nitrosative stress condition. This suggests a regulatory role for AtHMAD1 in NO biology.

## Author Contributions

QI, AH, B-WY designed the experiments QI, NF, AH, B-GM, AS executed the experiments S-UL, K-MK, AS analyzed the data, QI, AH, B-WY wrote the manuscript.

## Conflict of Interest Statement

The authors declare that the research was conducted in the absence of any commercial or financial relationships that could be construed as a potential conflict of interest.

The reviewer IBR and handling Editor declared their shared affiliation, and the handling Editor states that the process nevertheless met the standards of a fair and objective review.

## Footnotes

1 http://www.arabidopsis.org/

2 http://string-db.org/

3 http://geneontology.org/

4 http://arabidopsis.info/

5 www.photoshop.com

6 http://geneontology.org/

7 http://enzyme.expasy.org/
