# Supplementary material for: Nitric Oxide Responsive Heavy Metal-Associated Gene AtHMAD1 Contributes to Development and Disease Resistance in Arabidopsis thaliana
Source: Front Plant Sci. 2016 Nov 21;7:1712. doi: 10.3389/fpls.2016.01712 (PMC5116471; doi:10.3389/fpls.2016.01712)
Supplement: Supplementary file 1 [file Data_Sheet_1.docx]

Supplementary Material

Nitric oxide responsive heavy metal associated gene *AtHMAD1* regulates plant disease resistance in *Arabidopsis thaliana*

Qari Muhammad Imran^1,a^ Noreen Falak^1,a^, Adil Hussain^a,b^, Bong-Gyu Mun^a^, Arti Sharma^a^, Sang-Uk Lee^a^, Kyung-Min Kim^a^, Byung-Wook Yun^a^*

*** Correspondence:** Corresponding Author: [bwyun@knu.ac.kr](mailto:bwyun@knu.ac.kr)

# Supplementary Figures and Tables

## Supplementary Figures


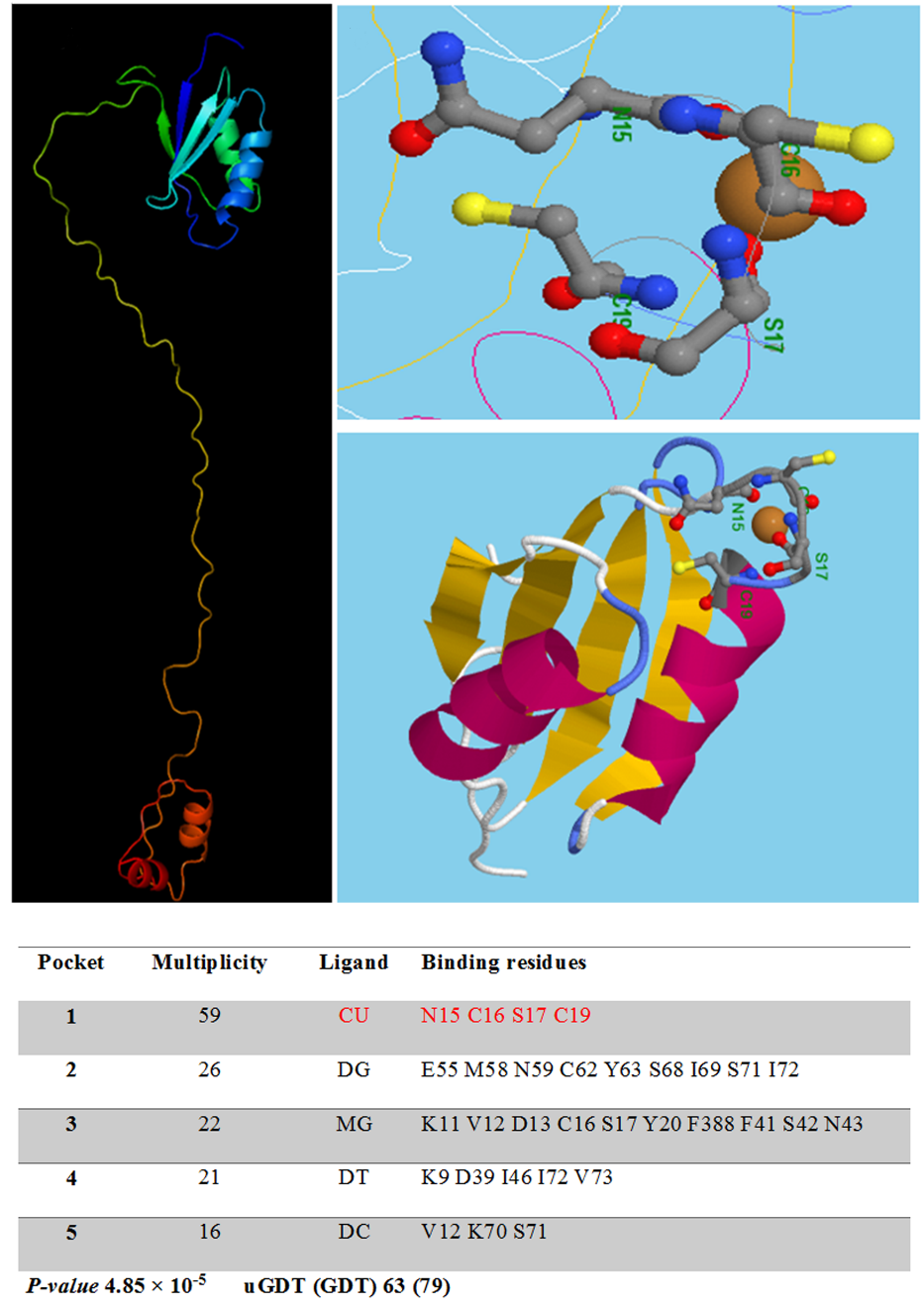


A

B

C

D


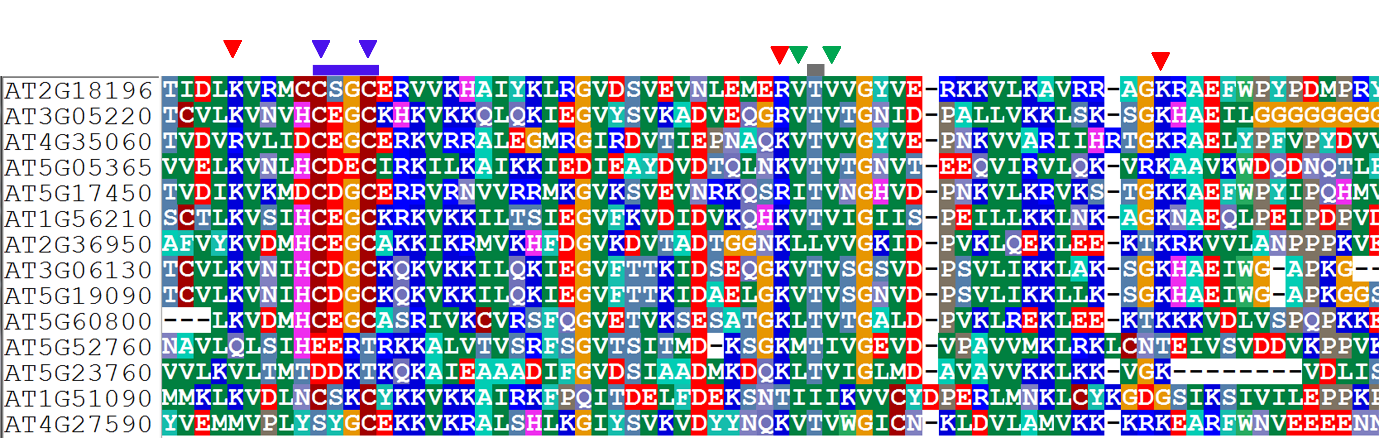


E

Supplementary Figure 1. Protein sequence and structure analysis of NO-responsive AtHMA gene products. (A) Complete 3D structure of interaction between AtHMAD1 and the Cu-ligand.ATHMAD1 showing both domains. (B) Close-up view of Cu-ligand. (C) 3D model showing interaction between Cu-ligand and AtHMAD1 at the designated amino acid residues (red color). (D) Table showing different residues predicted to bind with ATHMAD1 at designated amino acids. DG: deoxyguanosine-5′-monophosphate; MG: Magnesium ion; DT: thymidine-monophosphate; DC: 2′-deoxycytidine-5′-monophophate. The reliable binding prediction is for the CU residue (in red) with the highest pocket multiplicity (PM) (>40). This residue has a significantly high likelihood (*P-value* = 4.85 × 10^-5^) for binding to the protein domain over a set of randomly generated models for this domain. (E) Analysis for conserved HMA domain. Blue rectangle shows heavy metal domain with two closely spaced Cysteine residues.


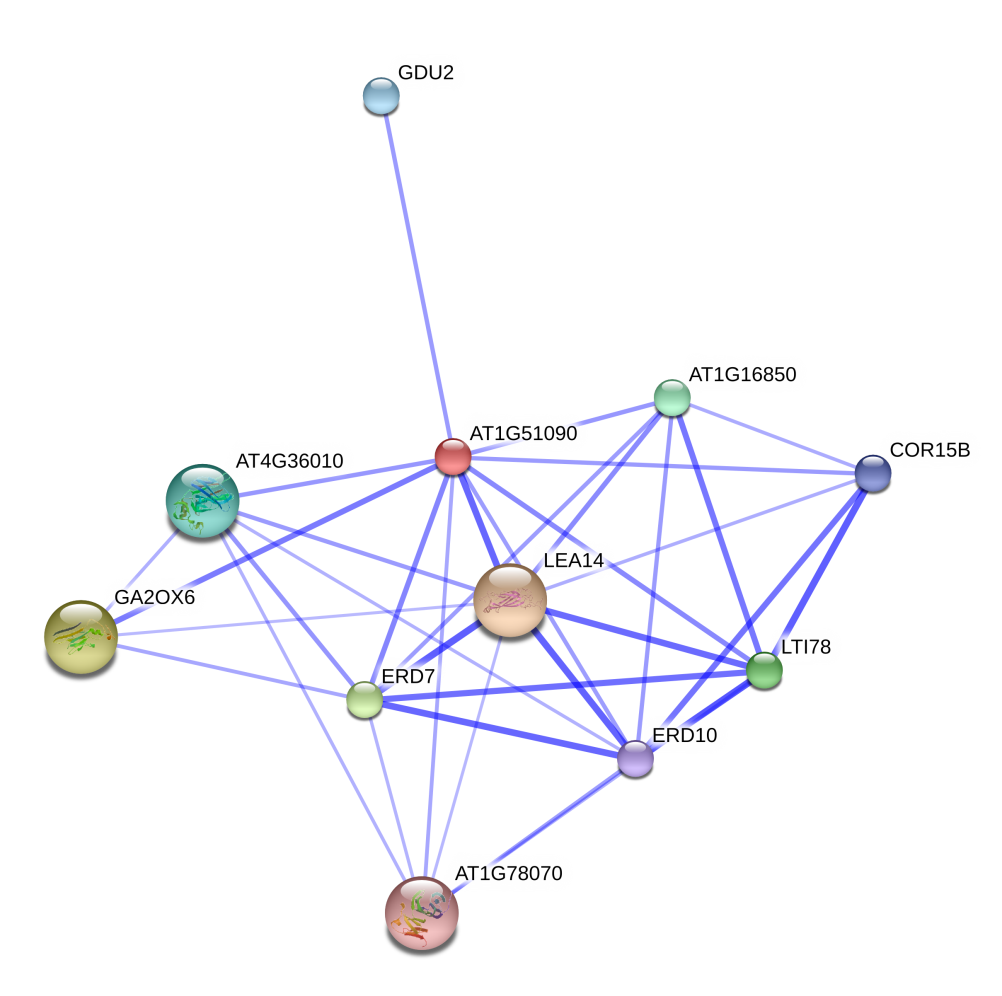


Supplementary Figure 2: *In silico* analysis of ATHMAD1 showing association with different proteins. The confidence view of protein-protein interactions. The stronger associations are represented by thicker lines.





Supplementary Figure 3: hypocotyl emergence of *athmad1* in response to the indicated stress conditions. The knock out mutant *athmad1* showed reduced hypocotyl emergence under all stress conditions studied. Error bars represents standard error (± SE, n=3). Asterisks represents significant difference compared to wild type.





Supplementary Figure 4: *Pdf1.2* expression in response to virulent *Pst* DC3000 at 5×10^5^ colony forming units concentration. . Error bars represents standard error (± SE, n=3).





Supplementary Figure 5: The induction of *Pdf1.2* expression after *Pst* DC3000 (avrB) at 5×10^5^ colony forming units. . Error bars represents standard error (± SE, n=3).





Supplementary Figure 6: The induction of *Pdf1.2* expression after *Pst* DC3000 (avrB) at 5×10^5^ colony forming units in systemic leaves. . Error bars represents standard error (± SE, n=3).

## Supplementary tables

Supplementary Table 1: Gene specific primer sequences for qPCR.

| S. No | Gene | Forward Primer Sequence | Reverse Primer Sequence |
| --- | --- | --- | --- |
| 1 | *AtPR2* | GTGCAATGGAGTTTGTGGTC | TCACATAATTCCCACGAGGA |
| 2 | *AtG3Pdh* | AAATATGTCGAGGCAAGGCT | CCACACAGCTTCTTGCAGAT |
| 3 | *AtPdf1.2* | CGCTGCTCTTGTTCTCTTTG | GGCTCCTTCAAGGTTAATGC |
| 4 | *AtActin2* | GCTGGACGTGACCTTACTGA | CCATCTCCTGCTCGTAGTCA |
| 5 | *AT1G51090* | TGTTCTTGTTTCGTCTTCCG | GTAAACGGGCCTTCCATAAC |
| 6 | *AT5G17450* | CCTGGCATGTACGATAAACG | GGAAACGATTGAGTGGGATT |
| 7 | *AT4G35060* | GCCAAATGCTCAGAAAGTGA | TACCGGTTCGGTGAATGATA |
| 8 | *AT4G27590* | TCTCGGACAAAGCCCTAGTT | TTCTGGATTCGCTGATTCTG |
| 9 | *AT5G52760* | AAGCCTGAACCTGAGAAACC | TTTGCATAGGCAGGATTGAA |
| 10 | *AT2G18196* | GAGGAAGAAGGTGCTCAAGG | GCGGGTGGTATCTTTGAAGT |
| 11 | *AT2G36950* | TTGGAGAGAAGAAAGCCGAT | ATCTTTAAAGGCACCACGCT |
| 12 | *AT5G23760* | AAATTGACGGTGATCGGTTT | CGGTCCAACCGATATCAAAT |

Supplementary Table 2: List of HMA domain containing genes that showed differential expression (up regulated= red color, down-regulated green color) to CysNO-mediated transcriptome analysis. FPKM(1) represents expression values before treatment while FPKM(2) represents expression values 6 h after 1mM CysNO treatment.

| S. No | Name | ^[[1]](#footnote-1)^FPKM(1) | FPKM(2) | Fold Change |
| --- | --- | --- | --- | --- |
| 1 | AT1G51090 | 2.51000 | 73.62000 | 4.87558 |
| 2 | AT2G18196 | 0.26000 | 4.03000 | 3.93271 |
|  | AT3G05220 | 18.46000 | 278.25000 | 3.91417 |
| 4 | AT4G35060 | 8.52000 | 43.64000 | 2.35621 |
| 5 | AT4G27590 | 0.13000 | 1.16000 | 3.17024 |
| 6 | AT5G05365 | 3.85000 | 20.90000 | 2.44134 |
| 7 | AT5G17450 | 6.39000 | 29.72000 | 2.21727 |
| 8 | AT5G52760 | 6.26000 | 18.24000 | 1.54329 |
| 9 | AT1G56210 | 4.23000 | 1.14000 | -1.89205 |
| 10 | AT2G36950 | 43.30000 | 20.84000 | -1.05497 |
| 11 | AT3G06130 | 8.34000 | 0.60000 | -3.80910 |
| 12 | AT5G19090 | 7.80000 | 1.25000 | -2.64179 |
| 13 | AT5G23760 | 60.76000 | 23.87000 | -1.34816 |
| 14 | AT5G60800 | 12.33000 | 3.64000 | -1.75981 |

1. Fragment Per Kilo Base pair [↑](#footnote-ref-1)
